# 56. A thirsty patient with painful joints and renal dysfunction

**DOI:** 10.1093/rap/rky034.019

**Published:** 2018-09-20

**Authors:** Kavina Shah, Shahir Hamdulay

**Affiliations:** Rheumatology, Northwick Park Hospital, Middlesex, UNITED KINGDOM

**Introduction:** We report a case of multi-system sarcoidosis with the rare manifestation of nephrogenic diabetes insipidus secondary to renal sarcoid demonstrated on histology.

**Case description:** A 52 year old lady was referred to rheumatology with polyarthralgia involving the shoulders, wrists and hands with morning stiffness and impaired fine motor function. Inflammatory markers were raised (ESR 70mm/h, CRP 17), with normal autoimmune screen and creatinine 70μmol/L (eGFR 81ml/min/1.73m^2^). X-rays of the hands, feet and chest were normal. She was diagnosed with a seronegative inflammatory arthritis and treated with sulfasalazine and prednisolone 7.5mg daily with good response. A year later she presented acutely with pleuritic chest pain, polyuria and weight loss. She was noted to have erythema nodosum and CT pulmonary angiogram excluded pulmonary embolus but demonstrated prominent mediastinal lymphadenopathy. Renal function was impaired (creatinine 141μmol/L) and serum ACE mildly raised (70). Calcium was normal (2.47mmol/L), urine dipstick negative for blood and protein, serum and urine protein electrophoresis negative and ultrasound of the renal tract normal. Diabetes mellitus was excluded with a normal fasting glucose (5.4) and HbA1c 41mmol/mol. A water deprivation test failed to concentrate urine nor improve with desmopressin indicating nephrogenic diabetes insipidus (DI). Subsequent renal biopsy demonstrated granulomatous tubulointerstitial nephritis, appearances consistent with sarcoid (image available but not able to upload to abstract submission). The patient was commenced on prednisolone 60mg daily and methotrexate with a rapid and marked improvement in symptoms and renal function (ESR 5mm/h, creatinine 93μmol/L and serum ACE 17).

**Discussion:** Sarcoidosis may present with an inflammatory arthritis, and up to one third of patients are reported to have musculoskeletal involvement. Up to 40% of patients may have renal disease. Hypercalcemia is the most prevalent cause of renal dysfunction in sarcoidosis followed by granulomatous interstitial nephritis (GIN), tubular dysfunction, obstructive uropathy, granulomatous angiitis and rarely glomerular disease. The true incidence of renal involvement in sarcoid is unknown, as is progression to chronic kidney disease. Hypercalcaemia and hypercalciuria is the predominant cause of renal disease in sarcoid, and if left untreated will result in progressive tubulointerstitial inflammation, nephrocalcinosis and CKD. Granulomatous interstitial nephritis was identified in 7-23% of patients with sarcoidosis in an autopsy study, although many were clinically silent. Nephrogenic DI in the context of sarcoid is an extremely unusual presentation. There are only two previous published reports, one in the setting of pulmonary sarcoidosis with DI due to hypercalcaemia in 2003, and another case from 1965 (normocalcaemic). Proposed treatment pathways for GIN in sarcoid include glucocorticoids, followed by azathioprine or mycophenolate mofetil, with the final step including biologic treatments including infliximab (3 previous case reports of using infliximab with positive outcomes). Transplantation has been reported in renal sarcoidosis, however in a French series of 18 patients, 17% had recurrence of disease, but the long term effect of this remains unknown.

**Key Learning Points:** We have described a case of seronegative inflammatory arthritis which predated the onset of more classical features of systemic sarcoidosis presenting with erythema nodosum, mediastinal lymphadenopathy and granulomatous tubuloinsterstitial nephritis with nephrogenic diabetes insipidus. In the setting of sarcoidosis, cranial DI is more common than nephrogenic DI, but both should be considered in sarcoid patients presenting with polyuria and polydipsia. Granulomatous interstitial nephritis is not uncommon in sarcoid however other conditions which mimic GIN must be excluded, which include allergic reactions, chronic fungal or mycobacterial infections and neoplasia. Serum ACE levels are increased in many patients with chronic kidney disease or end-stage renal disease of varying causes. The degree of elevation does not correlate with severity of the underlying renal failure. The majority of renal sarcoid is due to hypercalcaemia and hypercalciuria, and the true incidence of renal involvement in sarcoid is unknown as is the progression to CKD. Rheumatologists need to consider the renal manifestations of sarcoidosis when assessing these patients, particularly those with hypercalcemia, and should screen with 24 hour urinary calcium collections, renal ultrasound and consider referral to nephrologists.

**Disclosure: K. Shah:** None. **S. Hamdulay:** None.

